# Emotional Memory Moderates the Relationship Between Sigma Activity and Sleep-Related Improvement in Affect

**DOI:** 10.3389/fpsyg.2019.00500

**Published:** 2019-03-12

**Authors:** Bethany J. Jones, Ahren B. Fitzroy, Rebecca M. C. Spencer

**Affiliations:** ^1^Department of Psychological and Brain Sciences, University of Massachusetts, Amherst, MA, United States; ^2^Neuroscience and Behavior Program, University of Massachusetts, Amherst, MA, United States; ^3^Department of Psychology and Education, Mount Holyoke College, South Hadley, MA, United States

**Keywords:** sleep, emotional memory, affect, sigma, mood

## Abstract

Sleep is essential for regulating mood and affect, and it also consolidates emotional memories. The mechanisms underlying these effects may overlap. Here, we investigated whether the influence of sleep on affect may be moderated by emotional memory consolidation. Young adults viewed 45 negative and 45 neutral pictures before taking an afternoon nap measured with polysomnography. Following the nap period, participants viewed the same pictures intermixed with novel ones and indicated whether they remembered each picture. Affect was measured with the Positive and Negative Affect Schedule (PANAS) at baseline before the initial picture viewing task, immediately following the initial picture viewing task, and following the nap. The ratio of positive to negative affect declined over the task period and recovered over the nap period. When controlling for pre-nap affect, NREM sigma activity significantly predicted post-nap affect. Memory for negative pictures moderated this relationship such that a positive association between sigma activity and affect occurred when memory was low but not when memory was high. These results indicate that emotional memory consolidation influences the relationship between nap physiology and mood.

## Introduction

Sleep is important for multiple domains of emotional functioning. One such domain is the regulation of mood and affect. It is well known that sleep loss leads to impaired mood; both total sleep deprivation and sleep restriction have been linked to mood deficits (Pilcher and Huffcutt, 1996; Watling et al., 2017). Further, sleep disturbances and alterations are prevalent among those with mood disorders and may contribute to the development of these disorders (Meerlo et al., 2015; Murphy and Peterson, 2015). For example, major depressive disorder is marked by increased sleep latency and awakenings, decreased rapid eye movement (REM) sleep latency, increased REM density and REM sleep duration, and declines in slow wave sleep (SWS).

The mechanisms underlying the contribution of sleep to daily mood/affect in healthy individuals are not well understood, but REM sleep and SWS are both implicated by prior work. In healthy male adults who underwent two nights of normal sleep and two nights of sleep restriction, reduced REM sleep was associated with reduced functional connectivity between the medial prefrontal cortex and amygdala, which was in turn related to increased anxiety (Motomura et al., 2017). Collecting dream reports from healthy adults upon awakening from REM sleep indicated a decline in negative dream emotion throughout the night, which corresponded to an overnight reduction in negative mood (Cartwright et al., 1998). Slow wave sleep has also been linked to mood in healthy individuals. Compared to those undergoing restricted sleep, individuals undergoing forced awakenings had reduced SWS and positive mood, with the reduction in SWS mediating the reduction in positive mood (Finan et al., 2015). To our knowledge, no links between sleep spindles and mood have been reported in non-clinical samples. However, lower sleep spindle activity has been observed in individuals with anxiety and depression (Lopez et al., 2010; Wilhelm et al., 2017), suggesting that spindle activity may also be important for regulating mood.

In addition to regulating mood, sleep consolidates emotional memories (Tempesta et al., 2018). Compared to those who remain awake, participants who sleep perform better at emotional memory tasks. For example, participants who slept for 3 h immediately after reading negative text performed better on a memory test 4 years later compared to participants who stayed awake immediately after reading the texts (Wagner et al., 2006). Moreover, the consolidation of emotional memory by sleep has been linked to mechanisms also implicated in the effects of sleep on mood. Negative memory consolidation has been associated with REM sleep (Wagner et al., 2001; Nishida et al., 2009; Cairney et al., 2014), slow wave sleep (Groch et al., 2011; Cairney et al., 2014; Payne et al., 2015; Alger et al., 2018), and spindle activity (Kaestner et al., 2013; Alger et al., 2018). The shared mechanisms by which sleep influences mood and emotional memory may result in interactions between these influences. For example, sleep-related emotional memory consolidation induces plasticity within the ventromedial prefrontal cortex, a key mood-regulatory center (Nieuwenhuis and Takashima, 2011). Such plasticity may contribute to or impact the effect of sleep on mood.

Indeed, previous research suggests that emotional memory consolidation may influence the relationship between sleep and mood. We observed that when controlling for pre-sleep affect, percent of time spent in SWS during the night predicts morning affect (Jones et al., 2016). Specifically, more SWS was related to worse morning affect in young adults. This relationship was moderated by negative memory performance such that better post-sleep recognition for negative pictures was associated with a stronger negative relationship between SWS and morning affect.

The objective of the current study is to further investigate the influence of emotional memory consolidation on sleep-related change in affect. Here, we sought to determine the relationship between sleep physiology during a daytime nap and change in affect over the nap period. We further sought to determine whether negative memory performance would moderate any such relationship. Based on our previous findings, we hypothesized that SWS would be associated with worse affect upon wake and that higher negative memory performance would be associated with a stronger negative relationship between sleep and affect.

## Materials and Methods

### Participants

Data were collected from 50 young adults between 18 and 28 years of age (*M* = 20.94; *SD* = 2.29; 35 females). Participants had normal or corrected-to-normal vision and no history of neurological disease, sleep disorders, head injury, or use of medications known to affect sleep or cognitive function. Participants were instructed to refrain from alcohol, sleep at least 6 h the night before the experiment, wake up no later than 8:00 AM the morning of the experiment, and limit caffeine intake the day of the experiment. All participants were compensated with payment or course credit. Experimental procedures were approved by the University of Massachusetts, Amherst Institutional Review Board and written informed consent was obtained before the experiment.

Seven participants were excluded from all analyses for sleeping less than 45 min (half of a typical sleep cycle), and 1 participant was excluded due to multiple awakenings due to construction noise near the sleep lab. Thus, analyses of affect are based on 42 participants. Due to data loss, sleep stage scoring was not possible for 13 participants. Thus, sleep stage analyses are based on 29 participants. Four additional participants were excluded from sigma and delta activity analyses due to poor recording quality at electrode site F3 and/or F4 (where these measures were calculated), leaving 25 participants for these analyses. Finally, 2 multivariate outliers were excluded from moderation analyses, leaving 23 participants (see Data Analysis for outlier detection). Demographic information and variables of interest showed similar characteristics across these four subsamples (see Supplementary Table 1).

### Materials

Stimuli were 90 emotionally negative and 90 emotionally neutral pictures. The majority of stimuli were obtained from the International Affective Picture System (IAPS; Lang et al., 2005). The rest were from an in-house set and were chosen to match the IAPS pictures in content and emotionality (Baran et al., 2012). Based on normative data and previous work in our lab (Jones et al., 2016), negative pictures were moderate to high in arousal, and neutral pictures were low in arousal.

### Procedure

**FIGURE 1:**
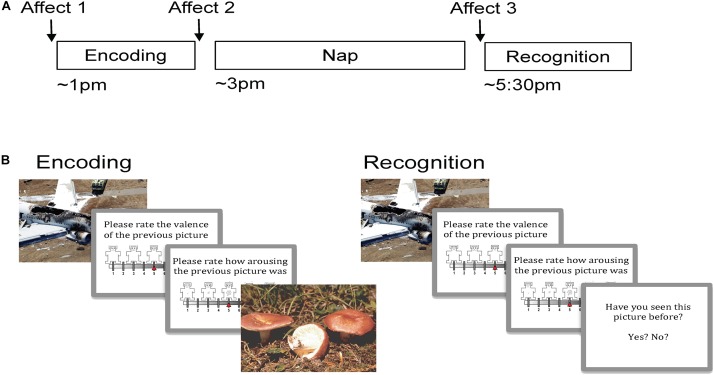
Experimental procedure and task. **(A)** Encoding took place in the early afternoon followed by a 2 h nap opportunity and then Recognition. Affect was measured prior to Encoding, just following Encoding, and then following the nap prior to Recognition. **(B)** During Encoding participants viewed 90 pictures (targets) and rated the valence and arousal of each on 9-point self-assessment manikin scales. During Recognition, participants viewed 180 pictures, a mixture of target and novel foil pictures, and rated each one on valence and arousal. Participants indicated whether or not they recognized the picture by responding yes/no.

Participants arrived for the Encoding session between 12:30 and 1:00 PM. Following Encoding, an electrode cap was applied and a 2-h nap opportunity was given. Following the nap opportunity, the electrode cap was removed and participants completed the Recognition session. Affect was measured at three time points: immediately before Encoding (pre-Encoding), immediately after Encoding (post-Encoding), and immediately before Recognition (post-nap; Figure 1A). Approximately 30 min passed between waking and Recognition to allow for dissipation of sleep inertia.

During Encoding, participants viewed 90 target stimuli (45 negative, 45 neutral) in random order (Figure 1B). Each picture appeared on the computer screen for 2 s, followed by a black screen for 6 s. After this black screen, participants were first prompted to rate the valence of the picture on a nine-item self-assessment manikin (SAM) valence scale (1 = negative, 5 = neutral, 9 = positive), and then prompted to rate its arousability on a nine-item SAM arousal scale (1 = no arousal, 9 = highly arousing; Bradley and Lang, 1994). Ratings were entered using numbers on a keyboard without any time limit. Following the rating scales, another black screen appeared for 10–14 s before the next picture. A long inter-stimulus interval was used in order to collect emotion physiology data (including skin conductance response), which are not presented here. Participants were not informed that their memory for the pictures would be tested later.

During Recognition, participants were shown 180 pictures: the same 90 targets seen during Encoding intermixed with 90 novel pictures (foils; 45 neutral and 45 negative). The stimulus presentation procedure was identical to Encoding with the following exceptions: (1) following valence and arousal ratings, participants were prompted to indicate whether they had seen each picture before by pressing “y” for yes and “n” for no, and (2) a 1-s inter-stimulus interval was used during the second half of the session in order to prevent fatigue.

### Polysomnography

Polysomnography (PSG) was recorded in the sleep laboratory using the Comet Plus PSG system (Grass Technologies) combined with a 32-electrode cap (EasyCap GmbH, Germany) that included two electrooculography (EOG; right and left ocular canthi), two chin electromyography (EMG), and 27 electroencephalography (EEG) leads (Fz, F3, F4, F7, F8, FCz, FC1, FC2, FC5, FC6, Cz, C3, C4, CP1, CP2, CP5, CP6, Pz, P3, P4, P7, P8, POz, O1, O2, M1, M2). PSG data were collected at a sampling rate of 200 Hz with a bandpass of 0.1–100 Hz. EOG and EEG channels were referenced to Cz during recording and re-referenced to the contralateral mastoid for scoring. Recordings were obtained and scored according to the specifications provided by the American Academy of Sleep Medicine (Iber et al., 2007).

### Data Analysis

Participants’ individual valence ratings of the pictures were used to categorize stimuli for analyses (as in St. Jacques et al., 2009). Due to individual differences in emotional response, individualized categorization may provide the most accurate measures. Targets were categorized based on ratings during the Encoding session, and foils were categorized based on ratings during the Recognition session. Negative and neutral pictures were defined as those rated 1–3 and 4–6 on valence, respectively. Hence, the analyzed picture sets were unique for each participant. On average, 36.92 ± 8.55 target pictures were rated as negative (valence: *M* = 1.83, *SD* = 0.53; arousal: *M* = 5.89, *SD* = 1.92) and 43.05 ± 11.60 target pictures were rated as neutral (valence: *M* = 5.02, *SD* = 0.13; arousal: *M* = 2.13, *SD* = 1.36). Hit rate, defined as the percentage of target pictures correctly identified as previously seen, was chosen as the memory measure based on our previous findings (Baran et al., 2012; Jones et al., 2016).

Affect was measured using the Positive and Negative Affect Schedule (PANAS; Watson et al., 1988). The PANAS consists of 10 positive and 10 negative attributes that participants rate on a scale from 1 to 5 according to their current feelings, resulting in a possible score of 10–50 for each valence. Higher scores indicate higher affect. Affect Ratio was calculated as an adjusted ratio of positive to negative affect for each of the three time points. Because a simple affect ratio (positive affect divided by negative affect) would range from 1 to 5 when positive affect is equal to or higher than negative, but would only range from 0.20 to 0.98 when negative affect is higher than positive, we calculated an adjusted ratio to keep negative and positive affect on the same scale. The adjusted ratio was calculated by dividing the higher valence score by the lower valence score, multiplying by -1 if negative affect was higher, and then subtracting 1 from positive values and adding 1 to negative values. This transformation resulted in a linearly spaced composite measure with a possible range of -4 to 4. Thus, positive scores indicate that positive affect was higher than negative affect, and negative scores indicate that negative affect was higher than positive affect.

EEG amplitude density was measured in the delta (0.5–4 Hz) and sigma (12–16 Hz) bands over frontal scalp regions (F3, F4) by extracting the amplitude envelope of bandpass-filtered EEG, summing it within identified sleep stages, and normalizing by time. The use of the Hilbert-transformation-derived amplitude envelope to quantify signal dynamics is a common method in engineering that has been previously applied to EEG analysis in multiple contexts, including sleep (Clochon et al., 1996; Freeman, 2004; Díaz et al., 2018). We opt to use this approach over the potentially more familiar short-time Fourier transform or wavelet decomposition methods of signal dynamics quantification because it is more computationally efficient, and because recent evidence suggests that Hilbert-transformation-derived envelopes may more accurately capture arrhythmic elements of the EEG (Díaz et al., 2018).

EEG data were first re-referenced offline to the averaged mastoid recording, then filtered separately into delta activity using a Butterworth infinite-impulse response filter (order = 2) that did not remove mean recording bias, and sigma activity using a forward impulse response filter (order = 164) that did remove mean recording bias. Regions of continuous filtered EEG exceeding frequency-band specific thresholds (delta: ±250 μV, sigma: ±75 μV) within a moving 500 ms window were marked as artifact. Delta and sigma amplitude envelopes were then calculated for each electrode as the magnitude (absolute value) of the analytic signal (z) of the filtered EEG, where the analytic signal is the sum of the filtered EEG and its discrete Hilbert transformation multiplied by the imaginary unit: z(EEG) = EEG + *i* ^∗^ Hilbert(EEG). Amplitude envelopes were then averaged across electrodes. Samples not previously marked as artifact at either electrode were then summed across stage 2 non-rapid eye movement (NREM2) sleep and SWS epochs, and divided by the combined number of artifact-free seconds spent in NREM2 sleep and SWS. Less than 0.04 and 0.02% of samples were marked as artifact for any participant for delta and sigma, respectively. EEG analyses were conducted in MATLAB using a combination of EEGLAB (Delorme and Makeig, 2004), ERPLAB (Lopez-Calderon and Luck, 2014), and custom in-house functions (available upon request).

Within-subject comparisons of means were conducted using repeated-measures analyses of variance (ANOVAs), and *post hoc* pairwise comparisons were made using Student’s paired-sample *t*-tests. Pearson’s *r* was used to assess bivariate linear relationships. Hierarchical multiple linear regression was used to conduct moderation analyses. Independent variables were mean-centered before being entered into regression models. Significant interactions were decomposed according to the guidelines of Aiken and West (1991), with fitted regression lines plotted at high (+1 *SD*) and low (-1 *SD*) levels of the moderating variable using estimates obtained from the final model. Simple slopes testing was conducted to assess relationships at high and low levels of the moderator. Multivariate outliers were detected and removed based on a studentized residual greater than 2.5 (1 data point removed) or a Cook’s Distance greater than 3 *SD* from the mean Cook’s Distance (1 data point removed). Significance levels were set to *p* < 0.05. A “marginal” effect was defined as having a *p*-value ≥0.05 and <0.075. Statistical analyses were conducted in SPSS, and interaction plots were created using open-source tools^1^.

## Results

### Change in Affect Over the Encoding and Nap Periods

**FIGURE 2:**
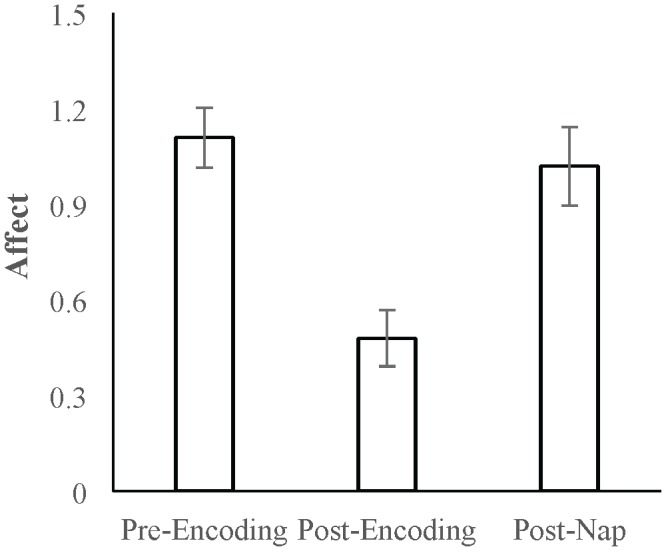
Mean Affect Ratio (adjusted ratio of positive to negative affect) at the pre-Encoding, post-Encoding, and post-nap time-points. Error bars represent standard errors of means.

Mean positive affect and negative affect scores measured at the three time points are reported in Table 1. A repeated-measures ANOVA with Time (pre-Encoding, post-Encoding, post-nap) as the within-subjects factor was conducted on Affect Ratio. There was a main effect of Time [*F*_(1.7,70.3)_ = 21.676, *p* < 0.001, Huynh-Feldt correction]. Follow-up paired-sample *t*-tests indicated that affect decreased over the encoding task period (*t* = 7.258, *p* < 0.001) and increased/recovered between the post-Encoding and post-nap time points (*t* = -5.670, *p* < 0.001; Figure 2).

**Table 1:** Affect [mean (*SE*)].

	Pre-Encoding	Post-Encoding	Post-Nap
Positive	25.24 (1.09)	18.07 (1.00)	21.90 (1.22)
Negative	12.19 (0.37)	12.36 (0.39)	11.12 (0.35)


We next analyzed positive and negative affect separately. For positive affect, there was a main effect of Time [*F*_(1.8,74.7)_ = 28.719, *p* < 0.001, Huynh-Feldt correction]. Follow-up comparisons indicated that positive affect decreased over the encoding task period (*t* = 8.717, *p* < 0.001) and increased/recovered over the nap period (*t* = -4.333, *p* < 0.001). For negative affect, there was also a main effect of Time [*F*(2,82) = 5.659, *p* = 0.005], with follow-up comparisons indicating that negative affect did not significantly change over the encoding task period (*t* = -0.382, *p* = 0.704) but did decrease over the nap period (*t* = 3.812, *p* < 0.001). Given that both positive and negative affect change over the nap period, we use Affect Ratio for subsequent analyses in order to capture overall affect while limiting the number of comparisons/tests.

### Relationships Between Nap Physiology and Affect

Average nap parameters are reported in Table 2. Of the 29 participants for whom sleep stage scoring was possible, 26 obtained SWS, and 16 obtained REM sleep. Nineteen participants were in NREM2 when they woke from the nap, 6 were in SWS, and 4 were in REM sleep.

**Table 2:** Nap characteristics [mean (*SE*)].

TST (min)	SL (min)	SE (%)	RL (min)	NREM1 (%)	NREM2 (%)	SWS (%)	REM (%)
93.34 (4.11)	12.40 (2.30)	80.68 (2.95)	63.61 (3.45)	11.66 (1.65)	50.01 (3.11)	25.59 (3.84)	12.75 (2.43)


*TST, total sleep time, SL, sleep latency, SE, sleep efficiency, RL, REM latency.*

**FIGURE 3:**
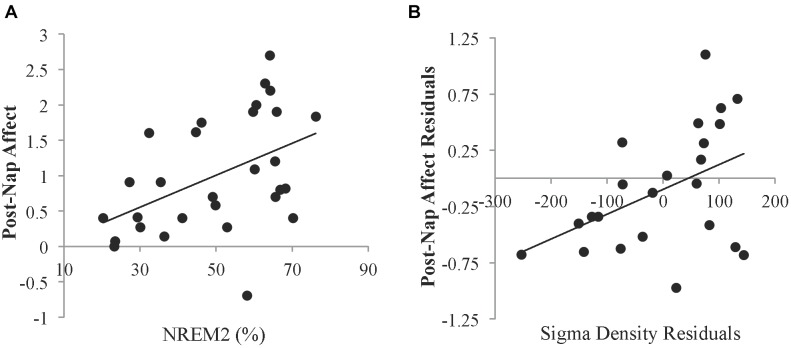
Relationships between sleep and affect. **(A)** Relationship between the percent of time spent in NREM2 sleep and the Affect Ratio (adjusted ratio of positive to negative affect) after the nap. **(B)** Partial correlation between sigma density and post-nap Affect Ratio when controlling for post-Encoding Affect Ratio. Residuals were obtained by regressing sigma density and post-nap Affect Ratio against the post-Encoding Affect Ratio. Sigma density values are reported in arbitrary amplitude envelope units summed per second. These values can be converted to mean amplitude envelope units (comparable to microvolts) by dividing by the sampling rate (200 Hz).

To investigate whether specific sleep stages were associated with the recovery in affect, correlation analyses were conducted. Neither percent time spent in SWS (*r* = -0.187, *p* = 0.331) nor REM sleep (*r* = -0.222, *p* = 0.247) was significantly related to post-nap affect. However, percent time spent in NREM2 sleep was positively related to post-nap affect (*r* = 0.459, *p* = 0.012; Figure 3A). Since sleep measures may be related to trait characteristics of affect, we next controlled for pre-nap (post-Encoding) affect to identify relationships with change in affect over the nap period. When controlling for post-Encoding affect, the relationship with percent time in NREM2 sleep remained marginally significant (partial *r* = 0.344, *p* = 0.073), suggesting that NREM2 sleep during the nap may be associated with improvement in affect. Neither SWS nor REM sleep was significantly related to post-nap affect when controlling for post-Encoding affect (*p*’s > 0.16). Furthermore, neither total sleep time nor sleep efficiency was significantly related to post-nap affect (*p*’s > 0.52). Post-nap affect also did not significantly vary according to the sleep stage from which participants awoke (*p*’s > 0.43), suggesting that individual differences in sleep inertia was not a factor in these results.

Given the relationship with NREM2 sleep, we investigated whether sigma activity (a hallmark of NREM2 sleep) was associated with improvement in affect. Controlling for post-Encoding affect, greater NREM sigma density was associated with higher post-nap affect (partial *r* = 0.439, *p* = 0.036; Figure 3B). To determine whether this relationship was specific to the sigma band, we also calculated delta density during NREM sleep. There was no significant relationship with delta activity during NREM sleep (partial *r* = -0.175, *p* = 0.414).

### Moderation by Memory

**FIGURE 4:**
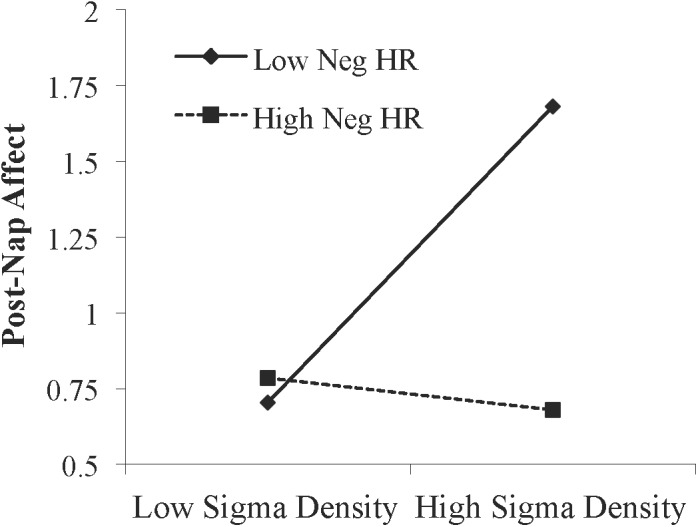
Interaction between NREM sigma density and negative memory in predicting the post-nap Affect Ratio (adjusted ratio of positive to negative affect), while controlling for the post-Encoding Affect Ratio. Neg HR, negative hit rate.

We next asked whether emotional memory consolidation influenced the relationship between sleep physiology and affect. Memory performance and relationships between memory and affect are reported in Supplementary Tables 1 and 2, respectively. A moderation analysis was conducted using hierarchical linear regression with post-Encoding affect entered in level 1 as the control variable, NREM sigma density and memory performance (hit rate for negative pictures) entered in level 2 as the predictor variable and moderator variable, respectively, and the NREM sigma density X memory performance interaction term entered in level 3. Sigma density (β = 0.259, *p* = 0.045) and memory performance (β = -0.289, *p* = 0.028) each predicted change in affect. Adding the interaction term significantly increased model fit, indicating that memory moderated the relationship between sigma density and improvement in affect (Table 3). Specifically, simple slopes testing indicated there was a positive relationship between NREM sigma density and change in affect at low (-1 *SD*) memory levels (β = 0.592, *p* = 0.003) but not high (+1 *SD*) memory levels (β = -0.090, *p* = 0.631; Figure 4). The moderation effect remained when we included false alarm rate as an additional control variable in the level 1 model (*R*^2^ change = 0.071, *p* = 0.024), suggesting that the effect is not driven by response bias. Additionally, the moderation was not significant using hit rate of neutral pictures (*R*^2^ change = 0.009, *p* = 0.445), indicating it is specific to negative memory.

**Table 3:** Multiple regression analysis.

	Post-Nap Affect		
Predictor	Model 1 – *B* (*SE*)	Model 2 – *B* (*SE*)	Model 3 – *B* (*SE*)
(Constant)	0.977 (0.117)^∗∗∗^	0.962 (0.098)^∗∗∗^	0.962 (0.087)^∗∗∗^
Post-Encoding affect	1.023 (0.201)^∗∗∗^	1.071 (0.170)^∗∗∗^	1.201 (0.160)^∗∗∗^
Sigma density		0.002 (0.001)^∗^	0.002 (0.001)^∗^
Negative hit rate		-2.692 (1.103)^∗^	-2.503 (0.979)^∗^
Interaction term			-0.027 (0.011)^∗^
*R*^2^	0.563^∗∗∗^	0.727^∗∗∗^	0.798^∗∗∗^
Δ*R*^2^		0.163^∗^	0.072^∗^


*B, unstandardized regression coefficient; *SE*, standard error; *R*^*2*^, model fit; Δ*R*^*2*^, change in model fit, ^∗^*p* < 0.05, ^∗∗∗^*p* < 0.001.*

## Discussion

Here we show that percent time spent in NREM2 sleep as well as NREM sigma density during an afternoon nap predict recovery of affect over the nap period following a decline related to viewing negative pictures. The relationship between sigma density and affect was moderated by memory performance for negative pictures. Specifically, this relationship was present for those with low memory performance but not for those with high memory performance. Further, this effect was specific to negative memory, as memory performance for neutral pictures did not moderate the relationship between sigma density and affect. These results may suggest that processing of emotional memories during sleep contributes to the impact of sleep on subsequent affect.

Based on our previous study of overnight sleep (Jones et al., 2016), we hypothesized that higher percent time in SWS would predict less improvement in affect over the nap. Instead, we did not find any significant relationship between SWS during a nap and affect. However, we did see that percent time in NREM2 sleep, as well as sigma activity [which is most prevalent in NREM2 sleep (De Gennaro and Ferrara, 2003)], were positively related to change in affect over the nap. Since percent time in NREM2 sleep and SWS are often inversely related, these findings may simply reflect this inverse relationship. Alternatively, there may be separate mechanisms acting on affect during NREM2 sleep and SWS, with a longer sleep period needed to uncover the relationship with SWS.

Sigma activity predominately reflects sleep spindles, though it should be kept in mind that they are not necessarily the same. While previous studies have more often linked REM sleep (Cartwright et al., 1998; Palagini et al., 2013; Motomura et al., 2017) and SWS/slow wave activity (Landsness et al., 2011; Cheng et al., 2015; Finan et al., 2015) to mood, some recent studies have implicated sleep spindles in relation to mood. Reduced spindle activity has been seen in children and adolescents with social anxiety, with greater fast spindle activity (13–16 Hz) related to less severe symptoms (Wilhelm et al., 2017). A reduction in spindle activity was also observed in children and adolescents with or at risk for depression (Lopez et al., 2010). In adults, reduced spindles have been reported in depressed individuals compared to controls (de Maertelaer et al., 1987), though there have also been reports of no difference between groups (Ferrarelli et al., 2007) or increased spindle activity in depressed individuals (Plante et al., 2013). Sleep spindles reflect synchronization between cortical and subcortical structures and promote synaptic plasticity, which may be integral to regulating structural and functional connectivity and effective communication among brain regions regulating mood and affect (Meerlo et al., 2015; Ulrich, 2016). Thus, sigma activity during sleep may generally benefit and restore mood. Lower trait-level sigma activity may predispose individuals to anxiety and mood disorders due to a reduction in the capacity of sleep to restore mood. Future research could investigate whether experimentally manipulating spindles could influence mood regulation.

In the current study negative (but not neutral) memory performance influenced the relationship between sigma activity and affect. Sigma density predicted improvement in affect only when memory performance was low and not when memory performance was high. These results are in some ways consistent with our prior findings. We previously observed a negative relationship between the percent time spent in SWS overnight and next morning affect (ratio of positive to negative affect; Jones et al., 2016). However, negative memory performance moderated this relationship. There was a significant negative relationship between SWS and affect only when negative memory was high, and not when it was low. Thus, in both the previous and current study, high negative memory was associated with worse affect than low negative memory. Since high memory performance suggests strong consolidation during sleep, these results may suggest that negative memory consolidation hinders the extent to which sleep benefits and restores mood.

Together, these current and past findings may suggest a multi-step process during sleep with regard to affect: sigma activity, most prevalent during NREM2 sleep, may benefit affect, but if subsequent mechanisms (particularly during SWS) lead to strong consolidation of negative memory (and thus high memory performance), affect is diminished. Thus, more sigma activity predicts better affect when negative memory is low, and more SWS predicts worse affect when negative memory is high. Additionally, we previously observed that high (but not low) *positive* memory performance was associated with a significant positive relationship between percent time spent in overnight SWS and morning affect in older adults (Jones et al., 2016). Thus, while consolidation of negative memories may adversely affect sleep-related restoration of mood, consolidation of positive memories may have the opposite effect. Since emotional memory consolidation involves mood-regulating circuitry, such as the ventromedial prefrontal cortex (Nieuwenhuis and Takashima, 2011), it may lead to functional changes within this circuitry that impact mood. More research manipulating memory valence and consolidation mechanisms such as slow wave activity is needed to investigate these possibilities.

This study provides evidence that emotional memory consolidation may impact the influence of sleep on mood. However, limitations of this research should be considered. First, these findings are associative in nature, and further research is needed to establish a causal relationship and determine the underlying mechanisms. Furthermore, though post-sleep memory performance is expected to reflect sleep-dependent memory consolidation to some extent, future studies should use over-sleep change in memory performance, as this change may be a more accurate representation of sleep-dependent consolidation. Finally, although fairly standard in the sleep and memory field, the sample sizes used in our sleep analyses (*n* = 23–29) are still relatively low, particularly for moderation analyses, and thus future studies with larger sample sizes are warranted.

## Data Availability

The raw data supporting the conclusions of this manuscript will be made available to a qualified researcher upon reasonable request.

## Author Contributions

BJ designed the experiments and collected the data. BJ and AF analyzed the data and wrote the manuscript. RS supervised the entire project.

## Conflict of Interest Statement

The authors declare that the research was conducted in the absence of any commercial or financial relationships that could be construed as a potential conflict of interest.

## Footnotes

1 www.jeremydawson.co.uk/slopes.htm
