# Supplementary material for: Emotional Memory Moderates the Relationship Between Sigma Activity and Sleep-Related Improvement in Affect
Source: Front Psychol. 2019 Mar 12;10:500. doi: 10.3389/fpsyg.2019.00500 (PMC6423070; doi:10.3389/fpsyg.2019.00500)
Supplement: Supplementary file 1 [file Table_1.docx]

**Supplementary Table 1. Subsample characteristics**

|  | **Affect Ratio**  **Sample**  **(*n*=42)** | **Sleep Stage Sample**  **(*n*=29)** | **Delta/Sigma Activity Sample**  **(*n*=25)** | **Regression**  **Sample**  **(*n*=23)** |
| --- | --- | --- | --- | --- |
| *Demographics* |  |  |  |  |
| Age (*M*(*SD*)) | 20.93 (2.22) | 20.97 (2.16) | 21.29 (2.19) | 21.17 (2.17) |
| Sex ratio (F:M) | 28:14 | 20:9 | 16:9 | 15:8 |
| *Measures (M(SE))* |  |  |  |  |
| Pre-Encoding affect | 1.11 (0.094) | 1.21 (0.111) | 1.31 (0.112) | 1.33 (0.121) |
| Post-Encoding affect | 0.48 (0.088) | 0.45 (0.115) | 0.53 (0.119) | 0.57 (0.124) |
| Post-nap affect | 1.02 (0.124) | 1.01 (0.153) | 1.04 (0.169) | 0.98 (0.168) |
| Negative HR | 0.85 (0.015)^a^ | 0.85 (0.017) | 0.87 (0.017) | 0.86 (0.018) |
| Negative FAR | 0.13 (0.011)^a^ | 0.13 (0.012) | 0.13 (0.014) | 0.13 (0.015) |
| Neutral HR | 0.80 (0.016)^a^ | 0.82 (0.017) | 0.83 (0.016) | 0.84 (0.016) |
| Neutral FAR | 0.17 (0.021)^a^ | 0.17 (0.027) | 0.16 (0.025) | 0.16 (0.027) |
| Delta density | -- | -- | 4109.94 (313.22)^b^ | 4169.71 (338.05)^b^ |
| Sigma density | -- | -- | 640.09 (21.434)^b^ | 635.97 (22.734)^b^ |

HR = hit rate; FAR = false alarm rate

^a^*n*=39 (memory measures not available for 2 participants due to data loss; 1 participant excluded due to poor performance (>3 *SD* below the mean))

^b^Delta and sigma density values are reported in arbitrary amplitude envelope units summed per second. These values can be converted to mean amplitude envelope units (comparable to microvolts) by dividing by the sampling rate (200 Hz).

**Supplementary Table 2. Correlations between memory and affect (Pearson’s *r*)**

|  | **Negative HR** | **Negative FAR** | **Neutral HR** | **Neutral FAR** |
| --- | --- | --- | --- | --- |
| *Affect Ratio sample (n=42)*^a^ |  |  |  |  |
| Post-Encoding affect | 0.262 | 0.081 | -0.061 | -0.064 |
| Post-nap affect | 0.090 | -0.046 | -0.265 | 0.096 |
| Change in affect^b^ | -0.115 | -0.133 | -0.300† | 0.184 |
| *Regression sample (n=23)* |  |  |  |  |
| Post-Encoding affect | 0.131 | -0.141 | -0.226 | -0.224 |
| Post-nap affect | -0.189 | -0.242 | -0.466* | -0.119 |
| Change in affect^b^ | -0.445* | -0.210 | -0.463* | 0.080 |

HR = hit rate; FAR = false alarm rate; † denotes 0.05<*p*<0.075; * denotes *p*<0.05

^a^Memory measures not available for 2 participants due to data loss; 1 participant excluded due to poor performance (>3 *SD* below the mean))

^b^Relationship with change in affect calculated as partial correlation between memory variable and post-nap affect while controlling for post-Encoding affect
